# Designing a Patient Preference Study on Subcutaneous Medical Devices: Incorporating Health Authority Scientific Advice and Patient Perspectives

**DOI:** 10.1007/s43441-024-00725-3

**Published:** 2025-02-26

**Authors:** Marie Picci, Nigel S. Cook, Byron Jones, Mo Zhou, Conny Berlin, Christine Sturchler, Clemence Martinez, Irene Garcia Baena, Lauren Ziegler, Harriet Gaunt, Brad Mason, Dominique Hamerlijnck, Yoshiyuki Majima

**Affiliations:** 1 Novartis Pharma AG, Basel, Switzerland; 2 Novartis Pharmaceuticals Corporation, East Hanover, NJ USA; 3 Adelphi Research, Bollington, Cheshire UK; 4 Adelphi Values Patient-Centered Outcomes, Bollington, Cheshire UK; 5 Atini, Amsterdam, Netherlands; 6 PanCAN, Chiyodaku, Japan

**Keywords:** Patient preferences, Patient experience data, Scientific advice, Subcutaneous delivery, Device features

## Abstract

This paper describes the planning of a patient preference study for evaluating device features for the subcutaneous (SC) delivery of high dose/large volume (e.g., > 2 ml) of drugs. Multiple sources, including qualitative patient interviews, the involvement of patient partners, and solicitation of advice from the US Food and Drugs Administration (FDA), were used to refine the attributes and levels in the development of a preference study protocol to investigate what Multiple Sclerosis (MS) patients consider important regarding medical device features for high dose SC administration.

**Supplementary Information:**

The online version contains supplementary material available at 10.1007/s43441-024-00725-3.

## Introduction

Patient experience data (PED) is increasingly important in regulatory and health technology assessment (HTA) decision-making [1]. A report commissioned by the FDA [2] identified good practices and areas for improvement in using PED for FDA decisions. Early and frequent consultation, data development, tool selection, data collection methods, and a strong data analysis plan were recognized as good practices. However, logistical challenges hinder timely FDA input on PED usage. An industry survey highlighted the need for expanded guidance and alignment to advance PED in FDA decisions [3].

Patient preferences are one type of PED [4], increasingly used to inform medical product decision-making. Large, multi-stakeholder initiatives such as the Medical Devices Innovation Consortium (MDIC) [5] and the Innovative Medicines Initiative (IMI) PREFER project [6] have developed guidance on when and how to elicit patient preferences to inform medical product decision-making. PREFER recommendations [6] and endorsement through the European Medicines Agency (EMA) qualification opinion [7], advocate early and iterative consultation meetings with regulators and HTA bodies in the form of scientific advice when the study concept is being finalized for the patient preference study (PPS) [6]. The PREFER Recommendations also advocate working with patients as research partners in the PPS to increase the relevance, appropriateness, feasibility, and acceptability of the study design, as well as its conduct and the interpretation of study results. Such multi-stakeholder alignment on the purpose and objectives of a PPS and on the design, conduct, and analysis is expected to lead to robust study results accepted by the different stakeholders to inform decision-making.

Currently, there are limited published examples of advice consultations with regulatory or HTA bodies for PPS design [8–21]. More examples are necessary to enhance learning, improve the process, and encourage others to seek advice and alignment during preference study design. It is also noted that although there has been significant growth in the involvement of patient partners in PPS design, this area still holds potential for further exploration and development.

This paper describes the preparation process for a PPS that evaluates device features for the SC delivery of high dose/large volume drugs. Multiple sources, including qualitative research studies, the involvement of patients as research partners, and scientific advice from the FDA, were used to refine the attributes and levels in the development of a preference study protocol to investigate what patients consider important regarding medical device features for high dose/large volume SC administration.

Preference studies have demonstrated the importance of SC device attributes as a factor in patient treatment choice [22, 23]. Matching device and treatment attributes to patients’ individual preferences has also been associated with increased treatment satisfaction, adherence, and improved health-related quality of life [24]. The present project seeks to inform the design of a PPS, and specifically the attributes and levels (A&L) grid for use in a discrete choice experiment (DCE), through engagement with different stakeholders and utilization of different qualitative research approaches. A PPS will increase the understanding of the acceptability, preferences and trade-offs made by patients, concerning device features and administration solutions for high dose/large volume SC delivery of drugs.

## Materials and Methods

### Overview of the Project Objectives and Research

The overall primary objective of this project is to quantify patient preferences and relative importance of attributes for different device and administration features to deliver high-dose/high-volumes SC formulations of treatment, in people living with MS in the USA. This objective was derived from FDA feedback on a quantitative PPS originally planned to be conducted as a “global “ DCE study with patients from several countries (USA, France, Germany, China, Japan) and across a variety of diseases areas (Oncology, Asthma). The initial design of the study was based on a targeted literature review and patient and health care provider (HCP) input from an initial qualitative study. Its objective was then reviewed and adapted following FDA advice through consultation meetings (Table 1).

**Table 1:** Summary of the key FDA advice provided for the PPS design through informal and formal consultation meetings.

FDA advice	Comments and actions taken and modifications to the study
Diversity in the patient population is to be expected: More focus on diversity by monitoring cultural diversity, including health literacy assessment, and measuring dexterity, physical limitation, health status and disease severity	The following updates to study design will be made: To providing an option to complete the survey in the Spanish language (in addition to English) in recognizing Spanish as the second most common language in the US Screening data to include demographic and cultural profiling metrics, including race, ethnicity, gender, age, income as socio-economic level, educational level, employment status, and insurance status Inclusion of questions on prior treatment history, patient characteristics, disease severity, current control of disease, physical limitation, dexterity, and comorbidities. Inclusion of health literacy assessment questionnaire Screening data will be monitored to ensure good diversity of study population, with ongoing recruitment adjusted based on these data
Eligibility criteria: Patients who do not have access to a mobile device, tablet, or laptop should not be excluded	Excluding participants who do not have access to a device, tablet, or laptop is a known limitation of online DCE studies when assuming that patients of lower socio-economic level may not have readily available or reliable access to these devices Through the collection of different demographics, efforts will be made to ensure participants of lower socio-economic levels are proportionately represented in the study, with recruitment being adjusted on an ongoing basis based on these data. The additional collected data (above) will help to complete the cultural profile of the study participants
Subgroup analysis by world region: Subgroup results per region (grouping countries) may be too heterogenous to draw a meaningful conclusion on preferences	The study team acknowledged this issue of known/potential cultural differences within a region drawing preferences. It was agreed to limit the country scope of the PPS to the US only
Disease areas and data transferability: MS patients are a different age-group than patients living with different types of cancer, with potentially different disease prognosis and timelines. Consider narrowing the disease scope	The study team acknowledged this feedback and decided to reduce the disease scope to only multiple sclerosis, but covering the three MS subtypes (RRMS, PPMS, SPMS) Since transferability of the PPS results to other indications such as non-small cell lung cancer, HER2 + breast cancer, chronic lymphocytic leukemia, or follicular lymphoma will no longer be explored within the revised PPS design, the study team acknowledged that additional research will be needed in the future to explore device preferences in these other patient groups
DCE analyses: Differences in preferences between subgroups should be tested first, before determining if aggregating data over the subgroups is possible	Reducing the scope to include only MS patients in the US will minimize the challenges raised when accounting for multiple subgroups in the DCE analysis. The study objective was modified to determine the patient preference and the relative importance of attributes for different device solutions, delivering high-dose/high-volume SC formulations of treatment (> 2 mL − 10 mL) to MS patients (RRMS, PPMS, SPMS) in the US The study will test for differences in preferences between subtypes of MS (RRMS vs combined SPMS + PPMS), prior to aggregation and further analysis of the full data set
Subgroup analyses: Sample size per subgroup should be sufficient to allow for analysis within a subgroup	A revised focus on MS patients only and the US only will allow for a more robust sample to be recruited in this study population (current target estimate of n = 280–360 MS patients: RRMS, n = 140; PPMS, n = 70–110; SPMS, n = 70–110)
Patient diagnosis / self-reported diagnosis: Patient self-reported diagnosis is not acceptable for FDA	The study team confirmed that confirmation of diagnosis via reliable sources will be conducted Recruitment through HCPs (estimated as 60% of the target sample) will collect evidence of diagnosis confirmed by HCPs For the remaining patients, recruiting via reliable sources such as patient advocacy groups, active patient panels, nurses, fellow patients and/or community leaders will ensure that participants have a verified diagnosis. For both recruitment channels, the screening questions will be designed to further verify the MS diagnosis
Need to obtain preliminary feedback from those populations with various disease severity to better assess the appropriateness of the attributes and levels	Additional qualitative research with MS patients in the US (conducted post-FDA meetings) was subsequently conducted to fully ensure study design and attributes and levels were optimized and appropriate for the MS population, including representation of RRMS, SPMS and PPMS patients
Alignment of the patient partner characteristics (disease area, disease severity) with the target quantitative study population	Four MS patients were added to the research team (Table 2) and involved in review of study materials developed

**Figure 1:**
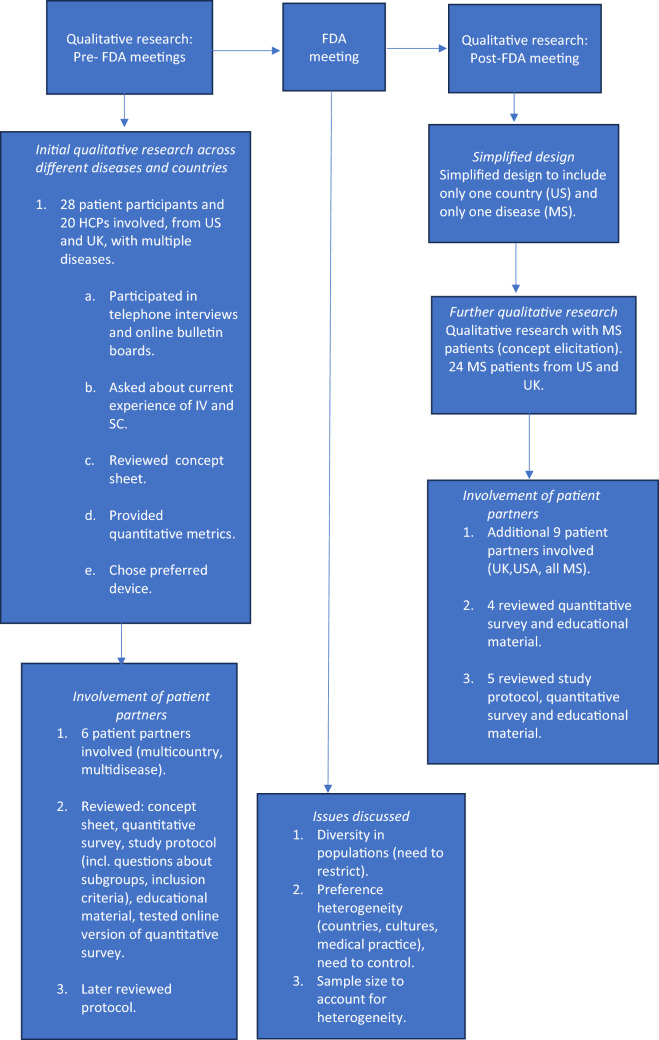
Main stages of preparatory qualitative work.

This final study was designed following a staggered approach using three steps (a. Pre-FDA meetings: Qualitative research, b. FDA meetings and c. Post-FDA meetings: Qualitative research) which are summarized in the flow chart given in Fig. 1 and further explained in Sections A to C below. The planned DCE study is now more specific in that it aims to gain a thorough understanding of the device preferences in one country and in one indication but will not support answering the question whether one device type might be a preferred option across multiple indications, which would require additional studies.

This article focuses on how initial qualitative research informed the development of an A&L grid, what feedback from scientific advice meetings with the FDA was critical in the study design, why additional in-depth qualitative research was necessary, and the importance and benefit of collaboration with patient partners in a preference study.

The execution and reporting of the completed DCE will be conducted as a next phase and its results will be published in a separate article when available.

### A. Pre-FDA Meetings: Qualitative Research

#### Initial Qualitative Research Across Different Diseases and Countries

Qualitative research was conducted in the USA and UK with 28 patients/caregivers and 20 HCPs to select and assess the importance of attributes related to SC injections. The questionnaire design for the qualitative research was informed through a prior targeted literature review based on patient experiences and needs regarding SC administration. This involved conducting telephone interviews with oncology, dermatology, respiratory and neurology patients and/or caregivers and with HCPs. The goal was to understand attributes of importance regarding the SC injection delivery, to gain insights into administration experiences, and elicit perspectives to hypothetical SC device concepts. The research sample recruitment by disease and specialty area is shown in Supplementary Material (SM) S1, and the discussion topics covered in the qualitative research are shown in SM S2. The respondents were asked to rate attributes related to SC injection by importance on a 0–10 numeric rating scale, with 0 being ‘not at all important’ and 10 being ‘most important’. Respondents were also asked to give their rationale for the ratings chosen.

The above findings were used to develop a “global” DCE study design presented to the FDA. The “global” study design originally included patients with different diseases (Oncology, Asthma) and different countries (USA, France, Germany, China, Japan).

### B. FDA Meetings

Novartis sought scientific advice from the FDA-CDRH PPI group (through the Q-Submission Program) on the “global” DCE, with the overall process from first contact and briefing book preparation to receipt of the final advice taking approximately six months. Two informal teleconference meetings were held prior to the formal request for a pre-submission meeting. These meetings were key to understanding general requirements on the submission route and introduce the overarching concept of the sponsor’s PPS to the FDA, noteworthy because of the very early stage in the device development when this interaction was taking place (in advance of any drug-device combinations or considerations of benefit/risk trade-offs). The first informal meeting's objective was to obtain guidance on the most expeditious submission pathway and appropriate FDA division from whom to request guidance on the PPS. The potential unmet medical need and rationale for the PPS in the complex context of a device development for potentially different diseases, key aspects of study design, details of the PPS objectives and planned application of the results were presented. After expressing Novartis’ intention to seek advice through the PPI group’s dedicated email (CDRH-PPI@fda.hhs.gov), FDA provided the opportunity for the first informal teleconference. A second (follow-up) informal meeting with the FDA PPI team was set up to continue the first meeting's discussions. Those informal meetings helped Novartis to better understand the FDA’s thinking and the FDA’s responses were a key driver for the team to refine the design quite significantly.

The timing of milestones during the advice procedure with the FDA are shown in Table 2. The briefing book content was informed by PREFER and FDA pre-submission guidance [6, 25]. The document included an introduction, a list of seven questions, covering three substantial topics in line with the recommendations of the FDA Q-Sub Guidance on the number of topics and lastly, background information on the project (see SM S3). The topics of discussion at the meeting and the subsequent FDA advice were in-line with the aspects of the PPS framework identified as particularly relevant for discussion during the scientific advice process, as described in the PREFER recommendations [6]. The key questions which were addressed with the FDA covered the following three substantial topics of (1) appropriateness of study purpose and intended use of the study results, (2) appropriateness of study design (study objectives, attributes and levels, patient centric study materials, statistical methods, sample size, participants’ diagnosis) and (3) transferability of the results to additional disease areas.

**Table 2:** Duration and milestones for FDA consultation on a Patient Preference Study for a device (CDRH Q-submission route).

	Project Milestone	Timing
1	Initial FDA contact, informal meetings, and briefing book preparation	3–4 months
2	FDA’s briefing book review and written feedback	70 days from submission
3	Scientific advice meeting	75 days from submission
4	Meeting minutes received	15 days from meeting

### C. Qualitative Research Post-FDA Meetings

Based on the discussions with FDA and their scientific advice, the design of the planned quantitative PPS was simplified to focus on one single disease (MS) and country (USA) instead of several indications across multiple countries. The updated study design allowed an increase in quality and robustness of the study results by focusing the study in one complex disease area (MS)/one diverse country and aligning the study population sample with the broader MS population including diversity in the MS patient demographics and characteristics. FDA recommended avoiding a broad scope of disease areas, since results may be too general and may have too many caveats to be useful in the end and may be more challenging to apply. The recommendation was to test the thinking in a specific area and then build from there to a broader scope. This required additional qualitative research to confirm the choice of attributes and levels in the PPS.

### Additional in-Depth Qualitative Research

**Figure 2:**
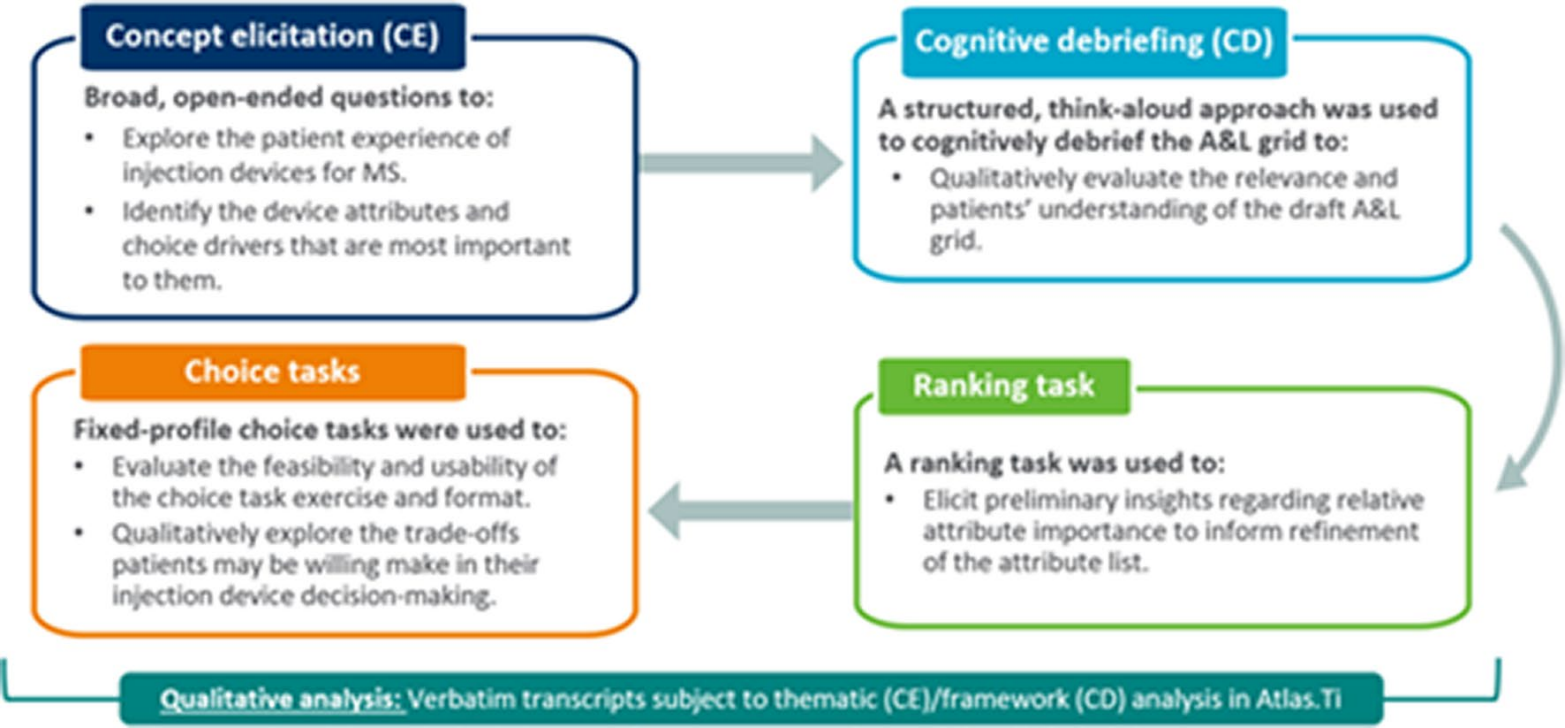
Structure of the concept elicitation and cognitive debriefing interviews.

Additional qualitative research was conducted to address FDA’s feedback. Twenty-four USA-based MS patients took part in an in-depth interview via telephone or screen-sharing software (Microsoft Teams). A combined concept elicitation (CE) and cognitive debriefing (CD) approach was taken (Fig. 2). The interviews aimed to generate evidence to inform A&L development and ensure the future PPS is fit-for-purpose in an MS patient population.

Concepts spontaneously discussed by MS patients were subject to concept saturation by the research team (see SM S4). Concept saturation is defined as ‘*the point at which no new concept-relevant information emerges*’ [26]. Concept saturation strengthens the study design by supporting the notion that enough interviews were conducted to be sufficient to capture the key treatment drivers of interest to MS patients. The FDA Patient-Focused Drug Development Series (specifically, FDA PFDD Guidance 2) [27] outlines guidance and agency expectations on using concept saturation to justify qualitative sample sizes. The interviews included participants across MS subtypes: Relapsing Remitting MS (RRMS), Secondary Progressive MS (SPMS), and Primary Progressive MS (PPMS). The sample size for each MS subtype was in accordance with concept saturation principles [28].

Each interview lasted approximately 60 min and was conducted by trained, experienced moderators who were briefed on population-specific considerations. Interviews were transcribed verbatim and subject to framework and thematic analysis methods [29–31]. An induction–abduction approach was taken to identify themes emerging directly from the data (inductive inference), and by applying prior knowledge (abductive inference).

The CE section involved asking broad, open-ended questions to encourage MS patients to discuss their treatment experiences and highlight features that are important when evaluating MS injection treatments.

The CD section used a ‘think-aloud’ approach [27, 32, 33] to assess consistency of understanding and interpretation, assess the relevance and importance of A&Ls selected, and confirm that appropriate framing (language and images) was used. The levels for each attribute were also explored to confirm if level differences were salient and differentiating. MS patients were asked to read aloud and discuss every A&L while responding to questions from the interviewer.

Study exemptions and approvals for the qualitative studies pre- and post-FDA meetings were granted by a centralized Institutional Review Board in the USA.

### A to C Involvement of Patient Partners

The research team worked in collaboration with patient partners on the research work pre- and post- FDA meetings as well as to prepare the Scientific Advice meeting with the FDA.

The patient partners were selected and were adapted as the project progressed to represent the target population in the different study designs. The rationale for involvement of patient panels for the “global” DCE study design pre-FDA consultation and MS DCE study design post-FDA consultation is described in Table 3. Patient partners were selected by the sponsor’s internal Patient Advocacy group in contact with patient organizations and communities. Key criteria for selecting patient partners included representativeness of the targeted disease population, ensuring a range of patient experiences, considering geographical representation, targeted characteristics like age or gender, cultural considerations, and English fluency (Table 3). Each patient partner was contracted individually and compensated for the time spent on the study, using the Novartis version of the Fair Market Value calculator developed by the National Health Council [34]. Individual on-boarding sessions with the patient partners were performed by the Patient Engagement representative and Project Lead to ensure sound understanding of the objectives of the PPS as well as their role as patient partners and related expectations. In addition, the Project Lead and Patient Engagement representative provided, during these on-boarding sessions, the objectives of the project beyond the PPS, the different activities performed in its scope and how the PPS will be used to inform this project. Interactions with the patient partners were conducted individually and virtually, at each key milestone of the study to collect their feedback, discuss and clarify any questions. Based on their feedback, the draft PPS documents were revised as required.

**Table 3:** Panel of patient partners prior to and following the FDA scientific advice consultation.

	Patient Partner	Country	Disease area of expertise	Rationale for involvement	Review
Panel prior to the FDA Consultation involved in the “global” DCE study design	1	Netherlands	Asthma	Research experience—input to the qualitative study phase	Concept sheet, study protocol and quantitative survey; educational material and test version of the online survey
	2	Japan	Pancreatic cancer	Administration device expertise	Concept sheet, study protocol and quantitative survey; educational material and test version of the online survey
	3	Germany	Breast cancer	Cancer support community including device experience	Study protocol; educational material and test version of the online survey
	4	Germany	Lung cancer	Experience with SC injection. Involved in previous Human Factor device study	Study protocol; educational material and test version of the online survey
	5	USA	Leukemia, Lymphoma	Cancer support community including device experience	Study protocol; educational material and test version of the online survey
	6	USA	Breast cancer	Input to the qualitative research study phase	Study protocol; educational material and test version of the online survey
Panel Post-FDA Consultation involved in the MS DCE study design	7	UK	RRMS	Expertise in clinical trials, device experience	Quantitative survey and educational material
	8	Germany	RRMS	Expertise in clinical trials, device experience	Quantitative survey and educational material
	9	UK	RRMS	Expertise in clinical trials, device experience	Quantitative survey and educational material
	10	UK	RRMS	Expertise in clinical trials, device experience	Quantitative survey and educational material
Additional patient partners with MS and from USA during the additional qualitative study and involved in the MS DCE study design	11	USA	RRMS	Patient Advocate for MS	Study protocol and quantitative survey; educational material video, educational material for qualitative study
	12	USA	RRMS	Patient Advocate for MS	Study protocol, and quantitative survey; educational material for qualitative study
	13	USA	RRMS	Expertise in device, health literacy	Study protocol and quantitative survey; educational material for qualitative study
	14	USA	PPMS	Expertise in clinical trials and device experience	Study protocol and quantitative survey; educational material for qualitative study
	15	USA	SPMS	Patient Advocate for MS	Study protocol and quantitative survey; educational material for qualitative study

*MS* Multiple sclerosis, *RRMS* Relapsing remitting multiple sclerosis, *PPMS* Primary progressive multiple sclerosis, *SPMS* Secondary progressive multiple sclerosis, *USA* United States of America, *UK* United Kingdom.

Patient partners helped to develop and review study material from its conception. The patient partners reviewed and provided comments on the concept sheet, giving an overview of the study design and device features, treatment process, attributes, and levels to be tested. The patient partners were then asked to review and comment on additional documents: the study protocol, quantitative survey script, Informed Consent Form, and educational materials to be provided to study participants (videos and infographics, as built into the study survey; see SM S5). Later, the patient partners were involved in reviewing and refining a test version of the online quantitative survey. The study team did not provide any guidance or specific direction as to which sections of the documents should be reviewed. Nevertheless, the Patient Engagement representative and Project Lead provided the PPS context in the project and were always available to connect with the patient partners, to ensure a clear understanding of the review expectations. Patient partners were also involved in reviewing the outputs of the initial qualitative research and FDA scientific advice feedback and providing context for the interpretation of the study results. Patients were not given a target number of hours to review the different documents. However, they were requested to carefully track the time spent on the review, for compliance and financial reasons. As an example, review of the draft protocol took a maximum of 10 h for a patient partner.

The feedback and comments from the above were used to develop the “global” DCE study design that was presented to the FDA as well as the DCE study design refined after the Scientific Advice with FDA. Following the FDA scientific advice, and the restriction of the PPS scope to MS patients and USA only, additional patient partners, who were MS patients living in the USA, were asked to review the revised study material.

## Results

### A. Qualitative Research pre-FDA Meetings and Input from Patient Partners for the Quantitative Study

The initial qualitative research approach blended qualitative in-depth telephone interviews with quantitative ratings, to gain a holistic view of the current perceptions of SC administration. Fieldwork was conducted between December 2021 and February 2022 in the USA and UK with 14 patients (a mix of oncology, dermatology, respiratory and neurology patients), each in the USA and UK, and with 10 HCPs each in the USA and UK (see SM S1).

**Figure 3:**
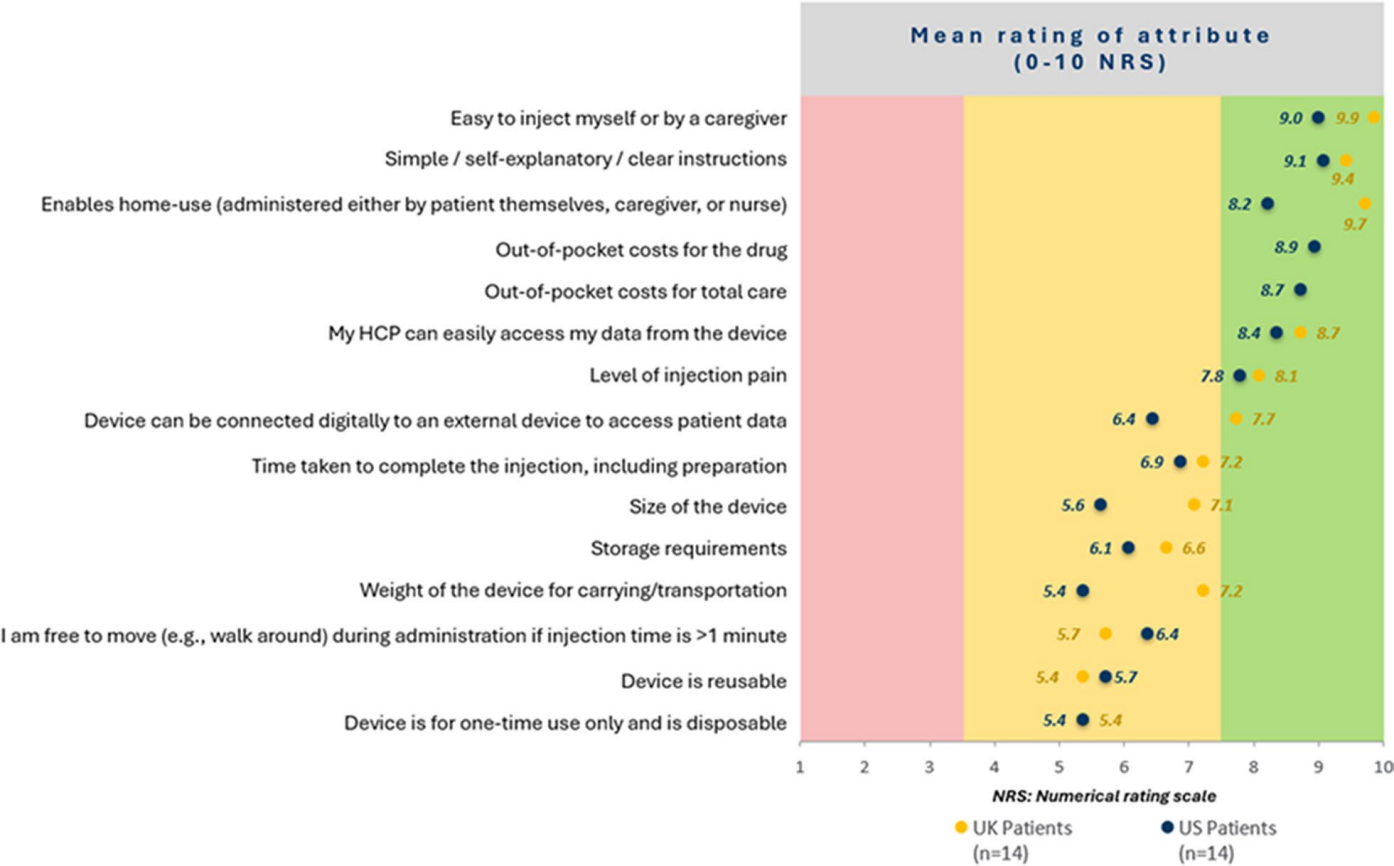
Ratings of importance of SC device attributes by patients/caregivers collected during the qualitative telephone interviews.

**Figure 4:**
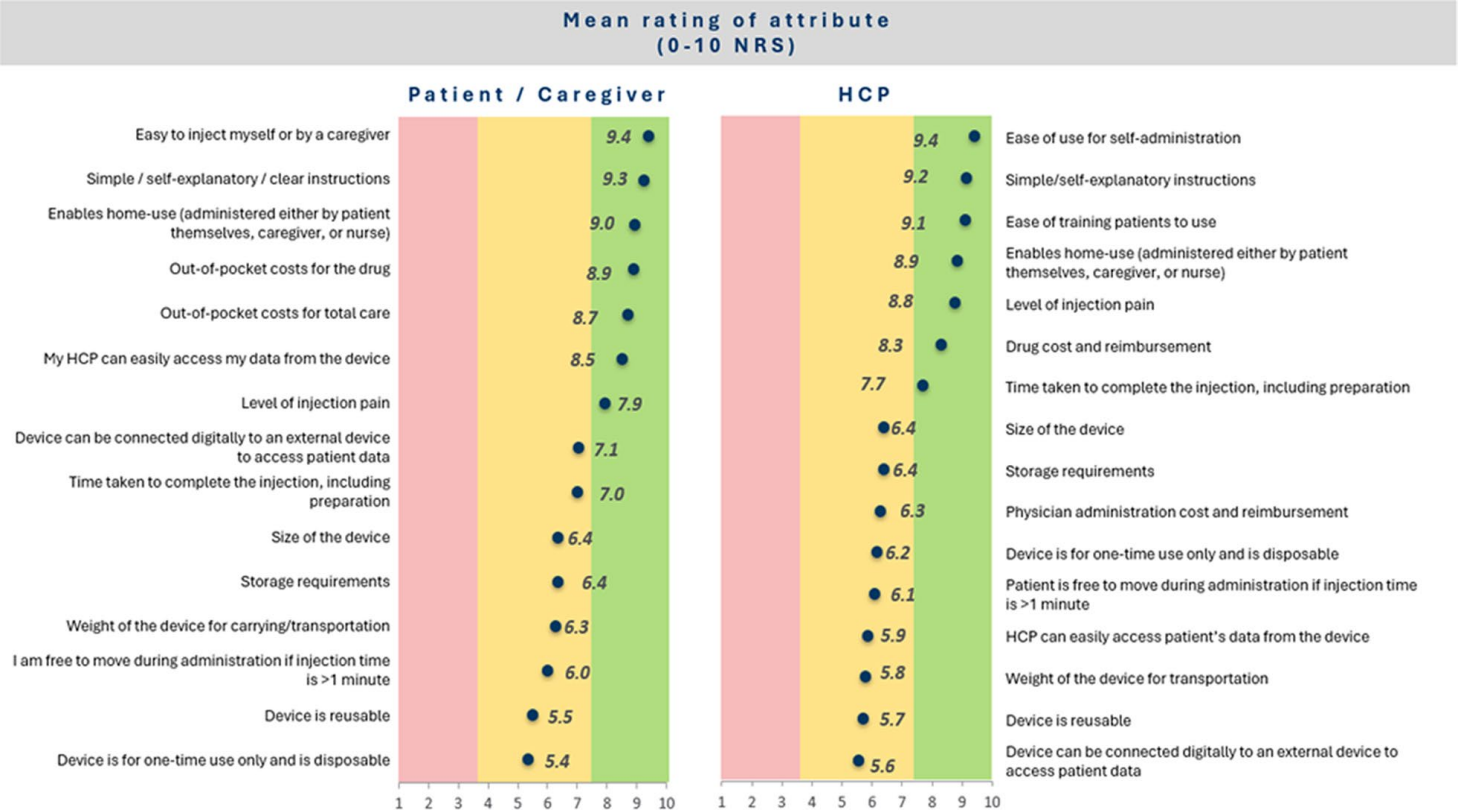
Ratings of importance of SC device attributes by patients/caregivers (left) and healthcare professionals (right). Aggregated data from US and UK (patients/caregivers, n = 28; HCPs, n = 20) collected during the qualitative telephone interviews.

**Figure 5:**
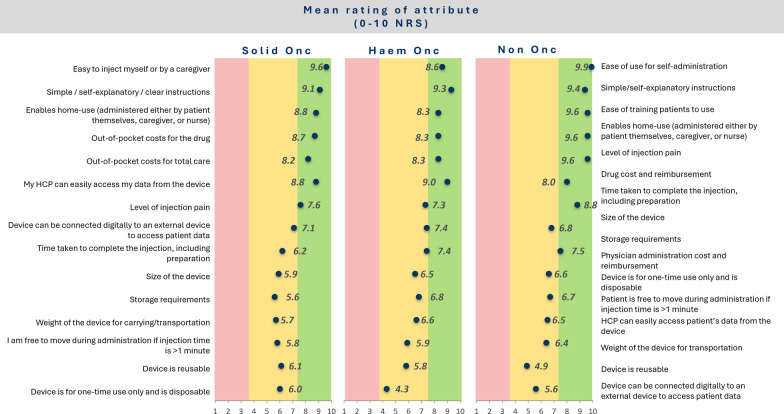
Rating of importance of SC device attributes by patients/caregivers. Aggregated data from US and UK (patients/caregivers), stratified by disease, collected during the qualitative telephone interview.

The results are summarized in Fig. 3. For patients, the three most important device characteristics were ease of injection, simple instructions, and having a device that enables home use. Patients from the UK and USA generally rated the importance of attributes consistently, while UK patients gave a higher rating to home use (9.7/10) than the USA patients (8.2/10). The USA patients gave a higher importance to total out-of-pocket costs of the treatment, total out-of-pocket costs for total care and HCP can easily access data from the device. Considering the aggregated results from UK and USA in Fig. 4, for HCPs, the first two highest rated attributes were the same as for the patients, with ease of training patients to use the device rated third. There was no strong evidence of a difference in the ratings across diseases (see Fig. 5).

In summary, both patients and HCPs welcome the idea of devices that allow SC delivery of a high dose/ large volume of drug and welcome a device that enables home use over hospital administration and injection timings for intravenous administration. The insights generated from this qualitative research were used to further develop the design considerations for a new high dose SC device and inform the attributes and levels of greatest importance to be explored further in the design of the quantitative PPS.

### Feedback from Patient Partners

The feedback from the patient partners on the initial qualitative study and used to prepare for the DCE is summarized in Table 4. It was highly insightful and led to revisions both to the study design and study materials. Key inputs of the patient partners that have led to improvements in the PPS design include: (1) conceptual remarks on the study design related to the inclusion/exclusion criteria and a proposal to revise certain sub-group analyses originally planned, (2) giving consideration to specific societal aspects in the study design (e.g., the potential impact of the recent COVID pandemic on the preference for home treatment) as well as potential sub-groups of interest (e.g., patient willingness to adopt a new technology, and potential differences in preferences between community areas (suburban/rural)). Suggestions were also made to simplify the language, add clarity to definitions and terms used, and improve the layout and educational material to facilitate clearer understanding by the study participants. Some of the feedback from the patient partners (Table 4) aligned with the comments the Sponsor later received from the FDA (Table 1).

**Table 4:** Feedback from patient partners pre- and post- FDA input.

Feedback on the “global” DCE study design prior to FDA input:	
Subgroups	Geographical subgroup: The patient partners who reviewed the Concept Sheet questioned the study team about the proposed subgroup analysis, particularly the geographic subgroup analysis (USA, Europe (France and Germany), and Asia (China and Japan)). They felt that the planned geographic subgroups lacked homogeneity because there are significant differences among them. For instance, the healthcare systems vary across different European countries, and China and Japan have distinct cultural perspectives on medicine. Including China and Japan in a single “Asian” subgroup, for example, would not enable a meaningful analysis. Consequently, China was removed from the study, to keep geographic subgroup analysis incl. USA, Europe and Japan New technology appetite subgroup: The patient partners who reviewed the protocol and quantitative survey script suggested adding in our analysis the level of appetite for new technology, because this may be an indicator for the willingness to adopt a new device technology. Thus, these subgroups were included in the analysis Community area subgroup: The patient partners who reviewed the protocol and quantitative survey script highlighted that the community areas of patients impact their preference of treatment ways (rural, sub-urban and urban). Consequently, these subgroups were included in the analysis Societal aspect: The patient partners shared that COVID pandemic may have influenced the treatment preferences for patients. Thus, a subgroup related to societal aspects has been included in the analysis
Inclusion criteria and disease characteristics	The patient partners raised concerns with the Sponsor’s proposals regarding the criteria for the self-injection experience. As a result, the Sponsor updated the inclusion criteria to include more specific details about the experience. Also, the PPS initially focused on examining disease severity's influence on device selection. However, the patient partners emphasized that factors unrelated to disease severity, such as dexterity or living area, may also affect the choice of treatment device. Therefore, additional characteristics were included for analysis

Feedback on the MS DCE study design post-FDA input	
Diversity inclusion	The patient partners provided feedback that the educational material and illustrations may not be representative of the targeted population for the PPS, as there was a lack of diversity in the representations. Consequently, the Sponsor adapted the educational material to be more inclusive, by adding different character genders, different skin colors in the pictures, different home depictions (see SM S7)
Colors and layout	Patient partners living with MS highlighted that the use of colors and color scale could be an issue for the Study participants living with MS, as they may have visual impairments. As a result, the Sponsor removed the colors and color scale from the answer scale (See SM S8)
Definition of terms/specific vocabulary	Several terms used in the questionnaire were still open to interpretation or too complex. The questionnaire has been revised to simplify the vocabulary used, especially the technical aspects of the devices, and new definitions have been added to ensure a good understanding by the PPS participants

Feedback on the additional qualitative study and MS DCE study design	
Layout	The layout of the educational material for qualitative study was confusing and the amount of text was increasing the complexity of reading and understanding. The same issue was present on the updated A&L grid, and text was reduced

### B. FDA Scientific Advice and Considerations for the PPS Study Design

The medical device PPS FDA CDRH pre-submission meeting and the two informal preliminary meetings with the FDA resulted in significant refinement of the study design to ensure a broad representation of the MS patient population, as summarized in Table 1. Most significantly, the study's scope was restricted to focusing only on MS patients and not to include patients from different disease areas in the target population (as originally intended). It was felt the different patient demographics and disease course could result in significantly different preferences across these indications, such that the planned sample size might not have allowed full examination of the differences. In addition, it was highlighted that differences between countries regarding culture, medical practice, and treatment standard of care, might also lead to preference heterogeneity (and require a much larger sample size to address this appropriately), leading to the decision to focus only on the USA for the DCE, with an increased MS patient target population size.

Other key learnings from the FDA advice included: ensuring sufficient diversity within the target patients recruited in the study and being representative of the overall USA population; ensuring sub-groups were of a sufficient size to allow meaningful analysis at the individual sub-group level, prior to aggregation of the overall study population for further analysis; inclusion of steps to ensure reliable diagnosis of the study respondents; and the need for additional qualitative research with the intended (MS) patient population to ensure appropriateness of the attributes and levels, prior to initiation of the DCE. All these FDA recommendations were addressed (Table 1) in the refinement of the DCE design and subsequent research as described in C, below.

Additional feedback was provided on the attribute *patient’s willingness to pay additional out-of-the pocket costs*, and FDA stated that preference on this attribute would not be used by FDA for decision-making in the scope of benefit-risk assessment. For that reason, it was decided to focus the preference study on the device features only and to consider patient’s willingness to pay additional out-of-pocket costs in separate cost-relevant discussions and decisions.

### Feedback from MS Patient Partners

Following the FDA scientific advice meeting, the updated A&L grid, study materials and DCE questionnaire were reviewed by MS patient partners. The feedback led to final small refinements of the study materials for the future full quantitative study in MS patients (Table 4).

### C. Additional Qualitative Research with MS Patients: Refinement of the A&L Grid for the DCE

In preparation for the DCE study, the A&L grid developed from the different feedback received from MS patient partners and FDA was pre-tested with MS patients in an additional qualitative study. This ensured the A&Ls were relevant in an MS population and allowed for any reframing of data collection questions in line with FDA draft guidance regarding diversity of recruited participants [35].

The tested A&L grid included six attributes in accordance with best practice for DCE studies [36]. The justification for attribute selection is summarized in SM S6.

**Figure 6:**
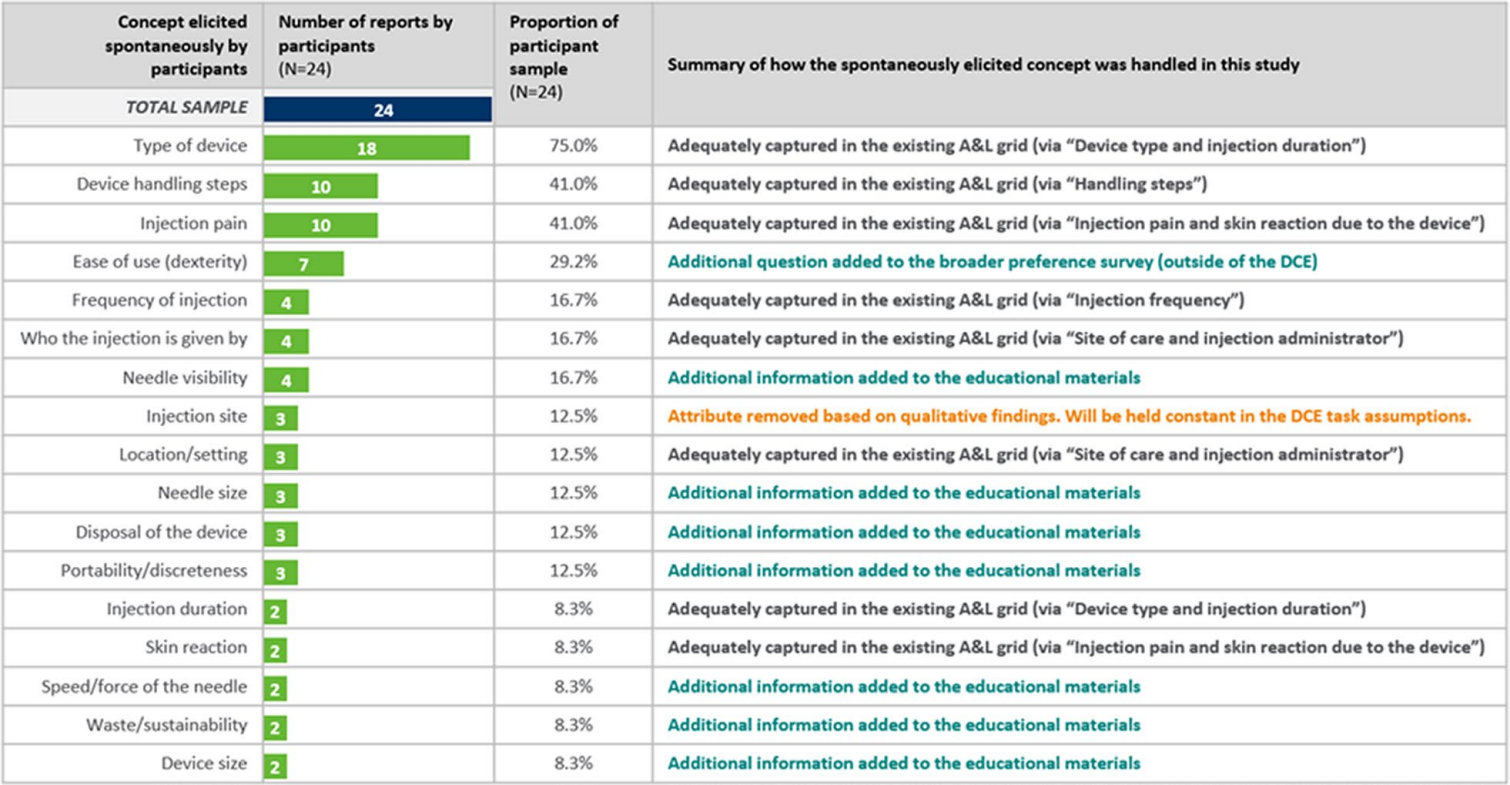
Spontaneous concept elicitation in US MS patients (n = 24).

The results of the CE questioning (Fig. 6) confirmed that the existing A&Ls selected for the DCE encompassed relevant concepts to MS patients in the context of medical device decision-making. Concepts spontaneously elicited in the CE questioning that were not deemed appropriate for inclusion as an attribute in the DCE were instead included in a general section of the questionnaire outside the DCE.

Concept saturation analyses indicated the sample size (N = 24) was sufficient to incorporate perspectives from a general, heterogeneous MS population.

**Figure 7:**
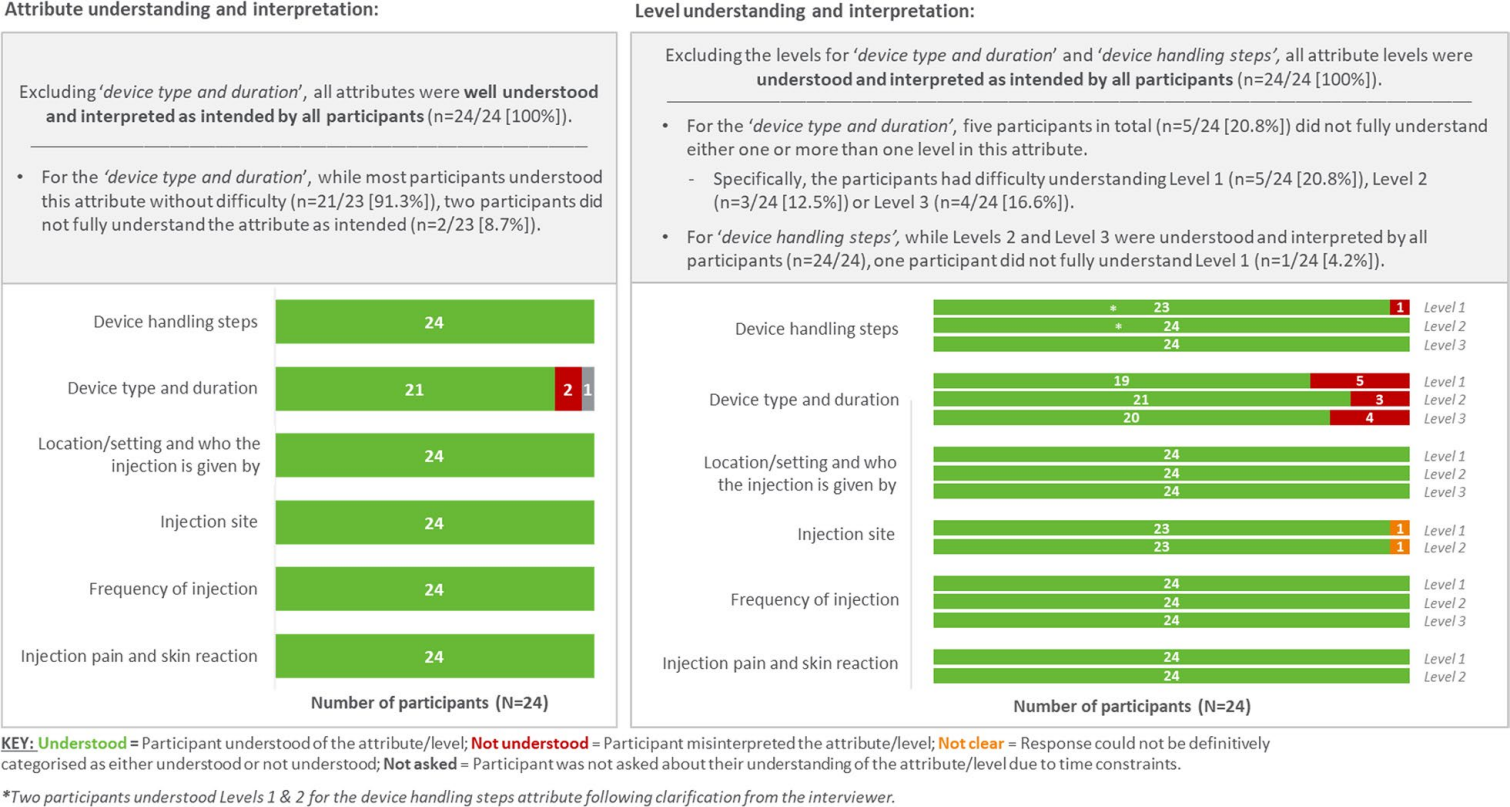
Cognitive debriefing of the A&L grid with MS patients in the US (n = 24). Assessment of attribute understanding of attributes (left) and of levels (right).

**Figure 8:**
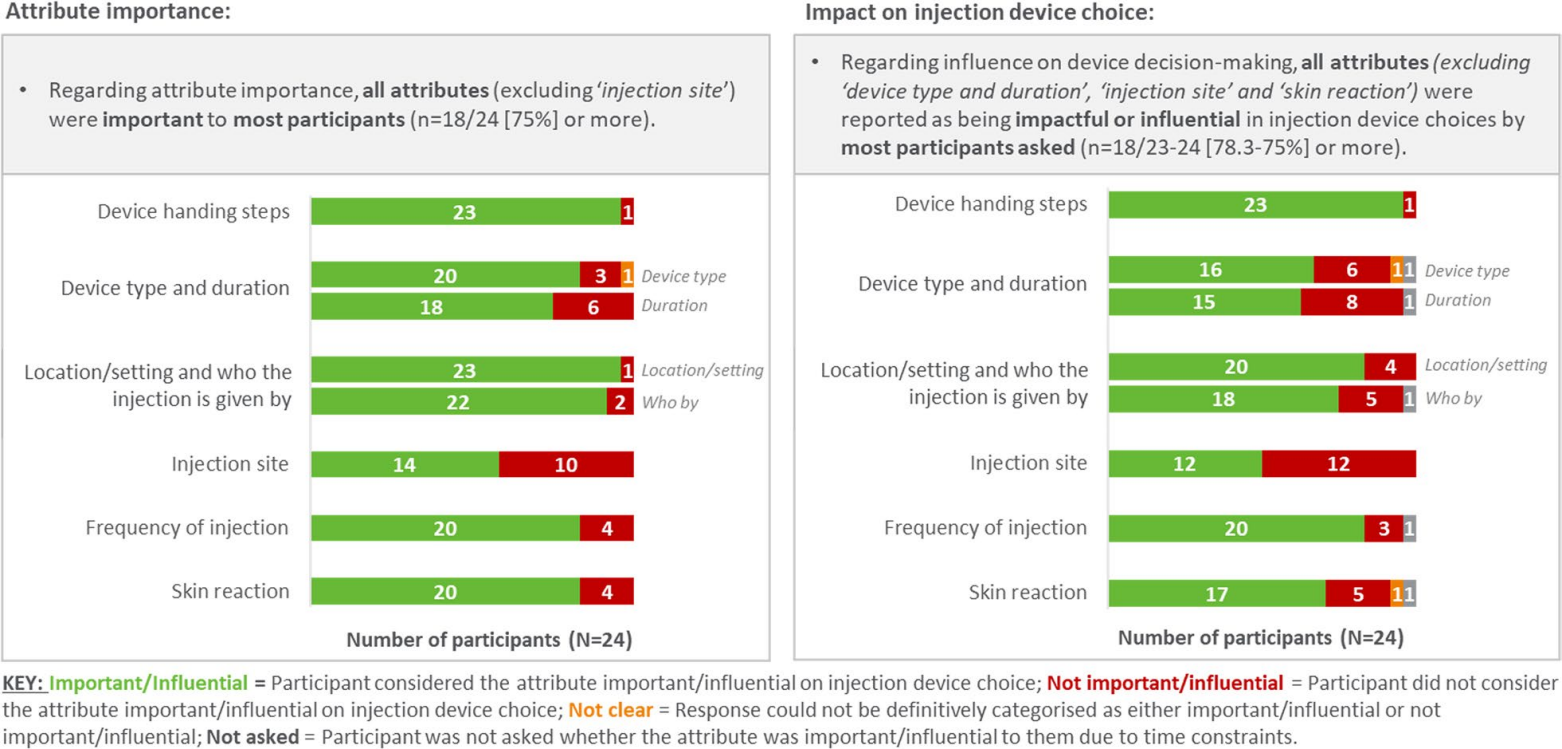
Cognitive debriefing of the A&L grid with MS patients in the US (n = 24). Assessment of attribute importance (left) and influence (right).

How well the MS patients understood the attributes and levels (displayed in Figs. 7 and 8) were assessed in the CD exercise using a think-aloud approach (Fig. 2). Figure 7 shows all attributes were well understood (87.5–100%). The individual levels were well understood for most attributes (95.8–100%), except for device type and duration (79.2–87.5%) where five patients had misunderstood one or more levels (12.5–20.8%). Figure 8 shows the attribute importance and impact on decision making: all attributes were important (75% or more) except injection site (58.3%). Figure 8 also shows that all attributes except injection site (50%), device type and duration (66.7% and 62.5%, respectively) and skin reaction (70.8%) were reported as being impactful or influential in injection device choice by most patients (75% or more).

The final A&L grid for the DCE (Table 5) took account of the qualitative feedback from MS patients regarding attribute-importance and impact or influence on device choice decision making (see SM S9).

**Table 5:**
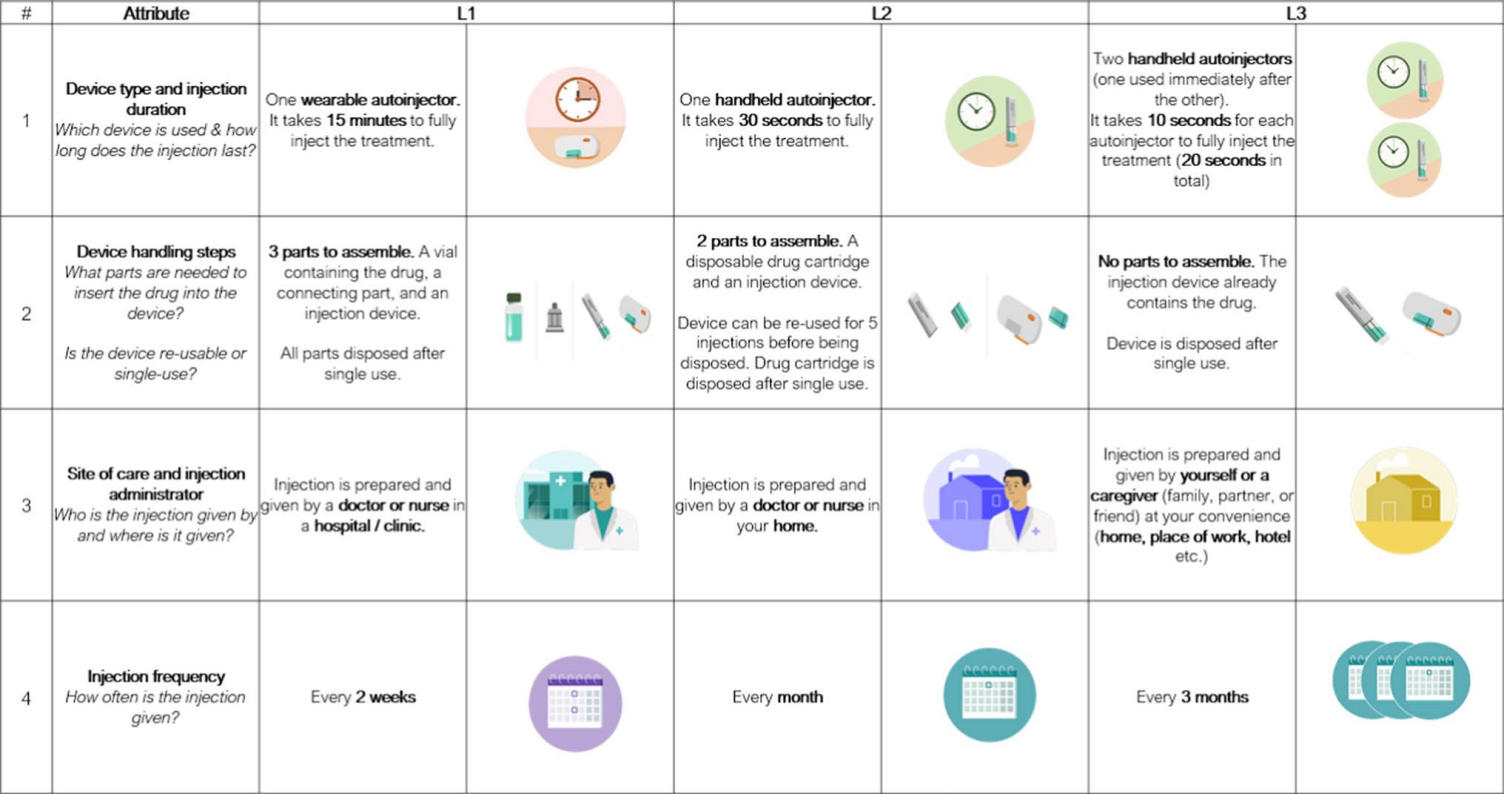
Final Attributes and Levels (A&L) grid.

Survey participants will be asked to assume the same drug efficacy and safety profile in all choice scenarios shown for different device options

### Feedback from MS Patient Partners

During the additional qualitative research, the patient partners were involved in reviewing the educational material used during the qualitative research as well as the updated A&L grid (Table 4). The educational material was updated to ease the reading and understanding by layout refinement. The feedback on the A&L grid led to final minor refinements.

## Discussion

This research project embraces several recently described best practices for designing a quantitative PPS, in particular the importance of high-quality qualitative research, involvement of patients as partners, conducting scientific advice with a health authority, and initiation of the research very early in the product lifecycle [2, 3, 6, 25, 35–43]. Our manuscript is one of the first examples to describe FDA scientific advice, how the process was conducted and how the input informed and improved the design of the PPS, building on the work of others [11, 12, 38] to illustrate the value created through this activity.

Early consultation supports the FDA’s recommendation of “early and often” dialogue between the Agency and study sponsors. Scientific advice ensures the design and methodology are fit for purpose and that studies meet Health Authority (HA) expectations, so that results can appropriately inform decision-making. To date, however, there are very few examples in the literature of the scientific advice /HA feedback process as it pertains to the design of a PPS: Tervonen and colleagues [11] have described a successful FDA Type C meeting process to inform the design of a DCE with alopecia areata patients, with changes made to the design based on the FDA feedback. Additionally, a recent conference panel discussion on maximizing the impact of patient experience data in regulatory decision-making, presented a case study where a PPS was designed to support benefit-risk trade-off discussions with the FDA on new product submissions, with a Type C and subsequent follow-up meetings held to inform the final preference study protocol and study design [12]. While the Type C meeting was considered successful, the process took 31 months, leading to recommendations for how the process could be improved in future. Two further examples of scientific advice regarding patient preference studies have been conducted with health technology assessment (HTA) bodies in Chronic Obstructive Pulmonary Disease (COPD) and Multiple Sclerosis [8–10].

Our scientific advice discussions with the FDA CDRH allowed the PPS design to evolve as an industry/patient/HA interaction. Such collaborative effort in this novel area of preference research is of utmost importance, where all stakeholders are learning and gaining knowledge about acceptable approaches to PPS design that ensure studies, and their results will meet the needs of decision-makers. In our experience, working with CDRH at FDA was smooth and was completed within six months. The initial informal discussions helped establish the information needed and format of the formal advice meeting, culminating in an efficient process and advice that was considered appropriate, timely, fit-for-purpose, and not prohibitive. This led to improved focus of the preference study design, which should enhance the richness of the study results. Whilst scientific advice on preference study design, especially advice sought very early in the product or device lifecycle, is still in its infancy and evolving as a concept, our experience was positive and will hopefully serve as a model that others can follow. It should be further noted that there is no dedicated meeting type for sponsors to discuss PPI with the FDA beyond that for medical devices. Guidance documents regularly recommend sponsors to contact FDA early and often but do not specify the appropriate meeting type for holding these discussions. Additional clarity regarding the appropriate meeting type would assist sponsors in planning patient preference studies and informing decision-making on whether and how to incorporate results into a development program. Although the project team did not face in this instance the logistical challenges described in the report contracted by the FDA [2], shorter advice timelines and an agile process to accommodate and promote medicine/device developers to pursue such studies, should be encouraged. To our knowledge, no other Agency offers a Scientific Advice process, specifically to fulfill the needs of Patient Preference Studies, which further reduces the opportunities of advice for PPS. More such examples are needed from other medicine and/or device developers working with decision-makers on PPS design, so that the advice process can be further optimized.

There is a recent evolution of the interaction between industry and the patient community [6, 44], going beyond the more traditional involvement of patients at specific touchpoints of the research, to engage patients as study members across the entirety of a project, as true research partners in all aspects of the research design, conduct and interpretation of results [1, 44]. Building on other recent best practice examples in this area [45] the observation that several of the recommendations made by patient partners in our study concerning design aspects and focus, were in alignment with advice obtained from the FDA through scientific advice, is a testament to the value created through their involvement. As discussed during the FDA advice meeting, the preferences of MS patients cannot automatically be assumed to represent the preferences of patients with other diseases (particularly those where characteristics of the disease and its course may differ greatly from MS) and thus, the additional engagement with people living with MS as patient partners post-FDA meetings helped refine the A&L grid of the future quantitative PPS.

Consistent with previous research [15–18, 23, 46, 47] the insights generated through the qualitative research with patients, caregivers, and HCPs, suggest an attraction toward easy-to-use devices for self-administration in the home setting. Ross et al. [22] have shown in a multi-country study, that independent, easy to perform self-injection were the most important device attributes for autoinjectors rated by both MS patients and nurses. Our quantitative DCE will further evaluate the relative importance of different SC device features (see Table 5) to MS patients, a population known to place high importance on the mode of treatment administration but also exhibit heterogeneity in their individual preferences [10].

Strengths of our study are based upon the comprehensive qualitative research using various explorative approaches, with a diverse representation of MS patients, to build a PPS design that is relevant and meaningful to the patient target group and should avoid uncertainties and misunderstandings when patients participate in the survey. The qualitative research supported the improvements to the survey study design (target groups, geography, statistical analyses) and informed the final DCE attributes and levels for the DCE. Comprehension of the DCE survey by participants will be ensured through the educational materials designed with the input of patient partners during these qualitative research phases. It will also serve as a template should there be interest in learning about the preferences of patients with other diseases in other countries.

As regards study limitations, the study is designed based on hypothetical devices and features in a virtual research environment, the patients having no physical contact with the devices in consideration.

## Conclusions

This research has resulted in the development of a study protocol for the investigation of importance to MS patients of medical device features for high dose SC administration, including the attributes and levels which will be central to the planned DCE. The research used a patient-centric co-creation approach, involving patients throughout as partners, in addition to the qualitative research conducted with patients, such that attributes and levels were developed that are relevant and comprehensible to patients. Input from a health authority through scientific advice, conducted very early in the development lifecycle, further ensures an optimal design for the PPS which is expected to lead to robust study results that will address stakeholder expectations and needs to inform decision-making. This supports the overall primary objective of the broader project, to determine with MS patients, the preferences and relative importance of attributes for different device and administration features, delivering high dose/high-volume SC formulations of treatment.

## Supplementary Information

Below is the link to the electronic supplementary material.

Supplementary file1 (DOCX 533 KB).

## Data Availability

No datasets were generated or analysed during the current study.
