# Supplementary material for: Designing a Patient Preference Study on Subcutaneous Medical Devices: Incorporating Health Authority Scientific Advice and Patient Perspectives
Source: Ther Innov Regul Sci. 2025 Feb 26;59(3):579–95. doi: 10.1007/s43441-024-00725-3 (PMC12018491; doi:10.1007/s43441-024-00725-3)
Supplement: Supplementary file 1 — Supplementary file1 (DOCX 533 KB). [file 43441_2024_725_MOESM1_ESM.docx]

**Designing a Patient Preference Study on Subcutaneous Medical Devices: Incorporating Health Authority Scientific Advice and Patient Perspectives**

**List of Authors:**

Marie Picci ^1^, Nigel S. Cook ^1^, Byron Jones ^1^, Mo Zhou ^2^, Conny Berlin ^1^, Christine Sturchler ^1^, Clemence Martinez ^1^, Irene Garcia Baena ^1^, Lauren Ziegler ^2^, Harriet Gaunt ^3^, Brad Mason ^4^, Dominique Hamerlijnck ^5^, Yoshiyuki Majima ^6^

**Supplementary Material (SM)**

###### **S1. Qualitative Research - Recruited sample for tele-depth telephone interviews (Patients, left, HCPs, right).**


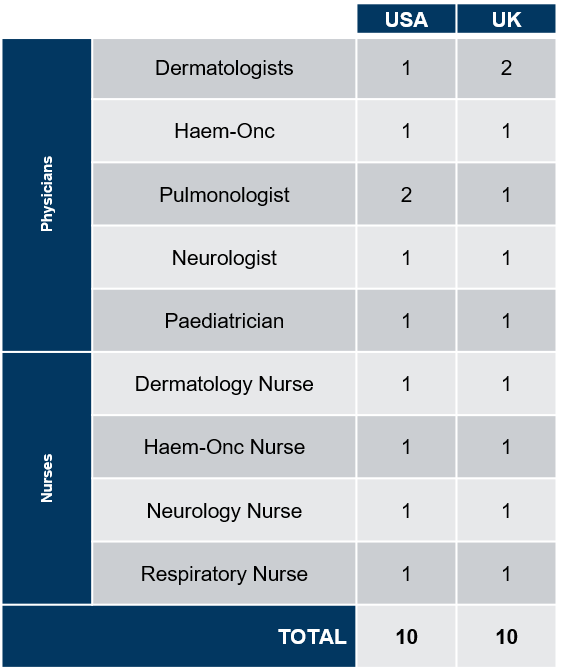

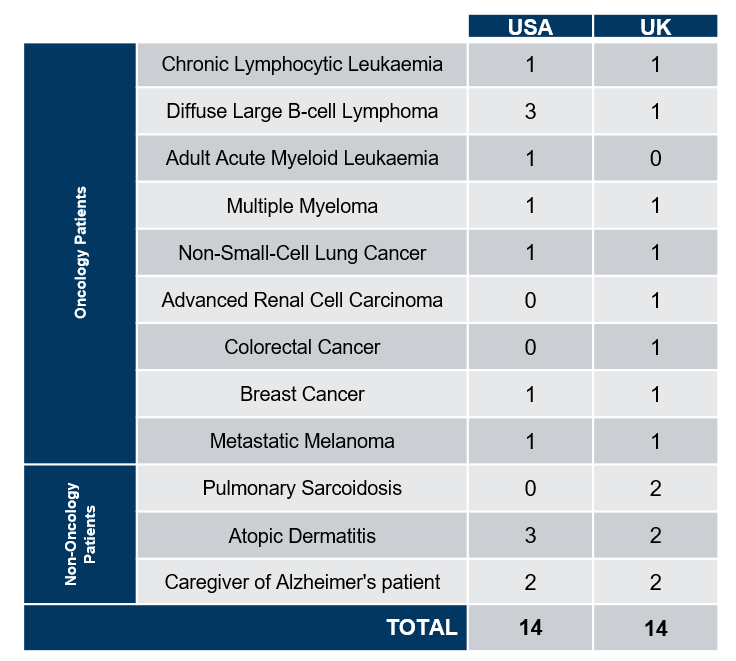


**Eligibility Criteria: Inclusion Criteria for Patients Participating in the interviews**:

- Patient must be over 18 years old.
- Patient must have been diagnosed with their condition by a healthcare professional.
- Patient must be currently receiving either a subcutaneous treatment or an intravenous treatment.
- The patient must have been diagnosed with their condition within the last 15 years.
- Patients must be comfortable discussing the benefits and drawbacks of treatment.

**Exclusion Criteria for Patients Participating in the interviews:**

- Patient must not be employed by or live with anyone employed by the pharmaceutical industry on a full-time or consultancy basis or employed by a communication or advertising industry related to pharmaceutical products.
- Patient is not willing to provide consent to take part in the research.
- Patient does not have access to a mobile phone or laptop to complete mobile ethnography.
- Patient is not able to understand and comprehend the study information.
- Patient has any other physical or mental illness that might influence the responses they give during the survey or might impact the patient’s ability to engage with the survey or provide appropriate input.

**Inclusion Criteria for Caregivers**:

- Caregiver must be over 18 years old.
- Caregivers must be a carer for someone with Alzheimer’s disease to take part in the study.
- Caregiver must be a carer for someone who has been diagnosed with Alzheimer’s disease by a healthcare professional.
- Caregiver must be currently caring for someone who receives either subcutaneous or intravenous treatment.
- Caregivers must be a carer for a patient with Alzheimer’s disease that has been diagnosed within the last 15 years.
- Caregiver must be comfortable discussing the benefits and drawbacks of treatment.

**Exclusion Criteria for Caregivers:**

- Caregiver must not be employed by or live with anyone employed by the pharmaceutical industry on a full-time or consultancy basis or employed by a communication or advertising industry related to pharmaceutical products.
- Caregiver is not willing to provide consent to take part in the research.
- Caregiver does not have access to a mobile phone or laptop to complete mobile ethnography
- Caregiver is not able to understand and comprehend the study information.

**Inclusion Criteria for HCPs and Nurses:**

- The HCPs and Nurses must be practicing in a clinical care setting for a minimum of 3 years.
- The HCPs and Nurses must see at least 10 patients a month that receive a subcutaneous or an intravenous injection and have an influence over treatment decisions.
- HCPs and Nurses must be practicing in an Office-based practice, Hospital-based practice or an Office and hospital-based practice.
- HCPs and Nurses must spend at least 70% of their time in direct patient care.
- HCPs and Nurses must be involved in either patient training, patient treatment education and assisting choice, or administering infusions.
- HCPs and Nurses must have experience of training patients on a wide range of devices and have the ability to recall devices for most common subcutaneous/intravenous injections spontaneously.
- Selected Nurses must be involved in discussions around treatment decisions with patients alongside their Primary Physician.

**Exclusion Criteria for HCPs and Nurses:**

- HCPs and nurses must not be employed by or live with anyone employed by the pharmaceutical industry on a full-time or consultancy basis, or employed by a communication or advertising industry related to pharmaceutical products.
- HCP or nurse is not willing to provide consent to take part in the research.
- HCP or nurse is not able to understand and comprehend the study information.

**S2. Discussion flow for the qualitative telephone research with patients and HCPs.**

The graphic below depicts the key stages of this study.

*Schematic of the Study Methodology – Patients / Caregivers*

*Schematic of the Study Methodology – HCPs*

Key topics covered during the qualitative interviews included:

- Current experience of both IV and SC administration routes
  - Functional and emotional journey.
  - Drivers and barriers across subcutaneous vs intravenous.
  - Current level of satisfaction.
  - Current unmet needs and support received.
- Concept review
  - Reactions to concepts for potential development.
  - Key considerations.
  - Anticipated level of impact.
  - Communication and support requirements.
- Quantitative Metrics
  - Multiple injections of short injection (3x 2mL) vs. large injection (e.g., 20 mins).
  - Pre-filled vs. not pre-filled (to be filled or to be assembled).
  - Single use vs. reusable device.
  - Injection time (seconds to 40 mins depending on volume).
  - Frequency of injection (daily, weekly, monthly).
  - Site of injection (abdomen, thigh, arm).
  - Location of drug administration (clinical and/or hospital).
  - Top 2 preferred concepts.

Interviews were conducted “live” via an online audio streaming platform, providing opportunity for identifying in real-time any key areas of interest for further probing, and recorded to facilitate analysis. A two-way screen allowed sharing of videos and stimuli with which the respondents could directly interact and for quantitative tasks to be completed during the interview.

###### **S3. FDA Briefing book content based on IMI PREFER and FDA pre-submission guidance.**

| **PPS Scientific Advice Briefing Book Section** | **Content** |
| --- | --- |
| Introduction | Background information on the disease and the product, unmet need, study rationale, previously requested scientific advice, development in other special populations, worldwide regulatory status of the product indicating timelines of marketing authorization application (MAA), Orphan designation of the product (if any), rationale for seeking scientific advice. |
| Substantial topics and questions | 1. Appropriateness of study purpose and intended use of the study results. 2. Appropriateness of study design    - Study objectives    - Attributes and Levels    - Patient-centric study materials    - Statistical methods    - Sample size. 3. Transferability of the results to additional disease areas. |
| Background Information | A sufficiently detailed overview regarding relevant clinical and non-clinical aspects of the molecule, the proposed PPS design, the overview of the involvement of patient partners, former qualitative research on patient preference and the limitations of the research methods and mitigation strategy (See A5.6 IMI PREFER recommendations).  The **Background Information** section covered the following PPS specific aspects:  Proposed Patient Preference Study design   - Research questionnaire and supporting study materials. - Ensure that the Questionnaire is in lay language and is patient focused. Confirmation of the patient-centricity of the survey should be included in this document. For more information, please refer to the Patient as Research Partners guideline. - Patient partners. - Include a section to provide visibility on the involvement of patients in the design of the study, a description of their involvement and the outcome and learnings from this interaction. This is for transparency purposes only and in-line with ICH E8(R1)14 Guideline and IMI PREFER recommendations. - Previous Qualitative Research. - This section should provide an overview of the literature search performed, a description of the qualitative phase, if applicable, and the findings driving potential decisions based on the proposed DCE. - Limitations of the research methods and mitigation measures. |

**S4. Concept saturation grid for the qualitative interviews conducted post-FDA meeting.**

|  | **Set 1** | | | | **Set 2** | | | | **Set 3** | | | | **Set 4** | | | | | **Set 5** | | | | **Set 6** | | | | | |  |
| --- | --- | --- | --- | --- | --- | --- | --- | --- | --- | --- | --- | --- | --- | --- | --- | --- | --- | --- | --- | --- | --- | --- | --- | --- | --- | --- | --- | --- |
| **Order of interviews** | 1 | 2 | 3 | 4 | 5 | 6 | 7 | 8 | 9 | 10 | 11 | 12 | 13 | 14 | 15 | | 16 | 17 | 18 | 19 | 20 | 21 | 22 | 23 | 24 | | |  |
| **Concepts elicited by participants that were captured in existing attributes in the A&L grid** | | | | | | | | | | | | | | | | | | | | | | | | | | | |  |
| Device handling steps | **S** | - | - | S | - | - | S | S | S | S | P | - | - | S | S | | - | P | - | - | P | S | - | S | S | | |  |
| Type of device | **S** | - | S | - | S | S | S | S | S | S | S | S | - | S | S | | S | S | - | S | - | S | - | S | S | | |  |
| Number of injections per dose | - | - | - | - | - | - | - | - | - | - | - | - | - | - | - | | - | - | - | - | - | - | - | - | - | | |  |
| How long an injection lasts for | - | P | **S** | P | P | - | P | - | P | - | - | - | - | - | - | | - | P | - | P | - | - | S | P | P | | |  |
| Where an injection is given | - | P | - | P | **S** | - | P | - | P | P | P | - | - | S | - | | P | P | - | S | P | S | P | - | - | | |  |
| Who the injection is given by | - | P | P | **S** | P | - | P | - | P | S | P | - | P | P | P | | P | P | - | P | P | P | P | P | S | | |  |
| Where the injection is given on the body | P | P | P | P | **S** | - | P | S | P | P | P | - | P | S | P | | P | S | P | P | P | P | P | P | P | | |  |
| How often the injection is given | - | **S** | P | P | P | - | P | P | P | P | P | - | P | P | P | | P | S | P | S | P | - | S | P | P | | |  |
| Injection pain | - | **S** | - | - | - | S | - | S | - | - | S | - | S | S | S | | - | - | S | S | S | - | S | - | - | | |  |
| Skin reaction | **S** | S | - | - | - | - | - | - | - | - | - | - | - | - | - | | - | - | - | S | S | - | - | - | - | | |  |
| **Concepts elicited that were not currently captured within existing attributes** | | | | | | | | | | | | | | | | | | | | | | | | | | | |  |
| Needle size | **S** | S |  |  |  |  |  |  |  | S |  |  |  | S |  | |  |  |  |  |  |  |  |  |  | | |  |
| Device size |  | **S** |  |  |  |  |  |  |  |  |  |  |  |  |  | |  |  | S |  | S |  |  |  |  | | |  |
| Disposal of the device |  |  |  | **S** |  |  |  |  |  |  |  |  | S |  | S | |  |  |  |  |  |  |  |  |  | | |  |
| Dose selector | **S** |  |  |  |  |  |  |  |  | S |  |  |  |  |  | |  |  |  |  |  |  |  |  |  | | |  |
| Pressure required to be applied to activation button on device |  |  | **S** |  |  |  |  |  |  |  |  |  |  |  |  | |  |  |  |  |  |  |  |  |  | | |  |
| Speed/force of needle |  |  | **S** |  |  | S |  |  |  |  |  |  |  |  |  | |  |  |  |  |  |  |  |  |  | | |  |
| Needle visibility |  |  |  |  |  |  |  | **S** | S |  |  |  |  |  |  | |  |  |  | S |  | S |  |  |  | | |  |
| Smart capabilities |  |  |  |  |  |  |  |  |  |  |  | **S** |  |  |  | |  |  |  |  |  |  |  |  |  | | |  |
| Ease of use (dexterity) |  |  |  |  |  |  |  |  |  |  |  | **S** |  |  |  | | S |  | S |  | S | S |  |  | S | | |  |
| Portability/discreteness |  |  |  |  |  |  |  |  |  |  |  | **S** |  |  | S | |  |  |  |  | S |  |  |  |  | | |  |
| Waste/sustainability | **S** |  |  |  |  | S |  |  | S |  |  | S |  |  | S | |  |  |  |  | S |  |  |  |  | | |  |
| Delivery of device to home |  |  |  |  |  |  |  |  |  |  |  |  |  |  |  | |  |  |  | **S** |  |  |  |  |  | | |  |
| *S: spontaneously reported, P: elicited once probed by interviewer, (-): not reported.*  *Highlighted S: the first time the concept was mentioned spontaneously* | | | | | | | | | | | | | | | |  | |  |  |  |  |  |  |  |  |  |  | |

###### **S5. Educational infographic material designed for the PPS, developed based on** **the qualitative research and patient partner input.**

| **Handling: Prefilled device**  **[HOVER OVER TEXT FOR BQ2a, BQ2b, BQ4 and DQ1]** | **Prefilled autoinjector** 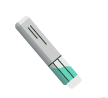  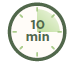  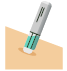  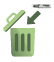 | **Prefilled patch injector**  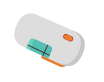  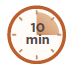  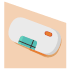  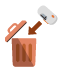 | This option is a device containing the treatment already.   1. After you remove the device from the refrigerator, you need to leave it out of the fridge for 10 minutes to allow it to warm up. 2. After 10 minutes, the device can be used, and the injection can be started. 3. The empty device is disposed of into a sharps bin after use. |
| --- | --- | --- | --- |
| **Handling: Reusable device** | **Reusable autoinjector**  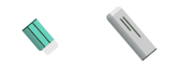  **Cartridge – device**  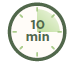  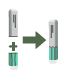  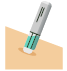  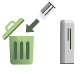 | **Reusable patch injector**  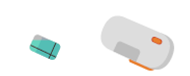  **Cartridge – device**  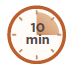  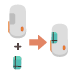  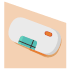  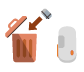 | This option includes 2 parts: a cartridge containing your treatment and a reusable device.   1. After you remove the cartridge containing your treatment from the refrigerator, you need to leave the cartridge out of the fridge for 10 minutes to allow it to warm up. 2. After 10 minutes, you can then insert the cartridge into the device. 3. The device is now ready to use, and the injection can be started 4. The empty cartridge is detached from the device and disposed of into a sharps bin after use. The reusable device can be re-used. |
| **Handling: Ready to fill device** | **Ready to fill autoinjector**  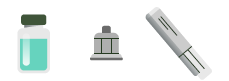  Vial – connecting part – injection device  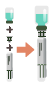  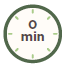  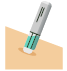  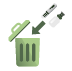 | **Ready to fill patch injector**  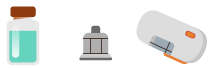  Vial – connecting part – injection device  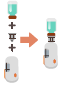  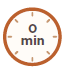  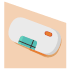  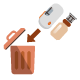 | This option includes 3 parts: a vial containing the treatment, a connecting part and a device.   1. After you remove the vial containing the treatment from the refrigerator, you attach the vial to the device with the help of the connecting part. The treatment is automatically transferred from the vial into the device. 2. No warm-up time is required.      1. Once the treatment is inside the device, you remove the empty vial and the connecting part from the device. The device is now ready to use and the injection can be started 2. All 3 parts are disposed of into a sharps bin after use |

###### **S6. Attribute justification**

| Attributes | Justification |
| --- | --- |
| **Handling steps**  *What are the device handling steps?* | Attribute influenced by usability, dexterity, disease type.  Most important attribute to patients and HCPs in the Qualitative study^1^: Easy to inject myself or by a caregiver; simple / self-explanatory / clear instructions. |
| **Device type and injection duration**  *Which device is used & how* *long the injection* *lasts?* | Attribute influenced by injection volume and injection skin reaction.  Top quartile most important attribute to patients and HCPs in the Qualitative study^1^: Time taken to complete the injection, including preparation. |
| **Site of care and injection administrator**  *Where and by whom is the injection given?* | Attribute influenced by usability (handling steps) and injection skin reaction.  Top quartile important attribute to patients and HCPs in the Qualitative study^1^: Enables home-use (administered either by patient themselves, caregiver, or nurse). |
| **Injection body site**  *Where is the injection given on your body?* | Attribute influenced by injection site reaction.  Injection body site limitation may exist for different diseases as per literature search^2^ and patient partners interview^3^ “Skin vulnerability is a well-known adverse event in a lot of disease areas and can be impacted by injection location”. |
| **Injection frequency**  *How often* *the injection is given?* | Attribute influenced by drug efficacy, injection volume and handling steps (reusable device rated equal to single used device by patients) ^1^.Less frequent administration preferred for SC injections of biologics, as per literature search^2,^ Patient Partners interview^3^ and as further confirmed in the in the Qualitative study^1^. |
| **Injection pain and skin reaction due to device**  *What is the injection pain and skin reaction caused by the device* | Attribute influenced by injection needle, speed, volume, body site and frequency and by adhesive patch.  Skin vulnerability – many patches create a reaction on the skin that can be quite severe for some patients, as per Patient Partners interview^3^ |
|  |  |

^1^ High dose SC devices qualitative research

^2^ Understanding and Minimising Injection-Site Pain Following Subcutaneous Administration of Biologics, Anja St Clair-Jones 2020

^3^ Patient Partners interviews: 2021 06 28 NOVARTIS High dose subcutaneous solutions; Patient Advisor Consultation Questions: Completed: July 6, 2021

**S7. Example of more inclusive imagery used in survey following review.**


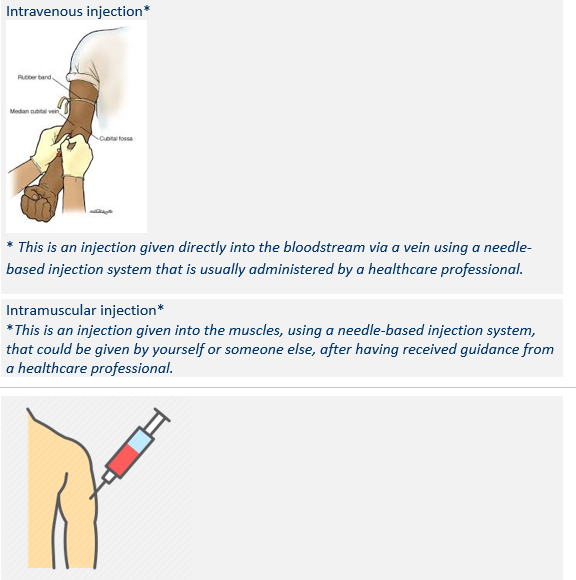


**S8. Color scale used in survey following patient partner review.**


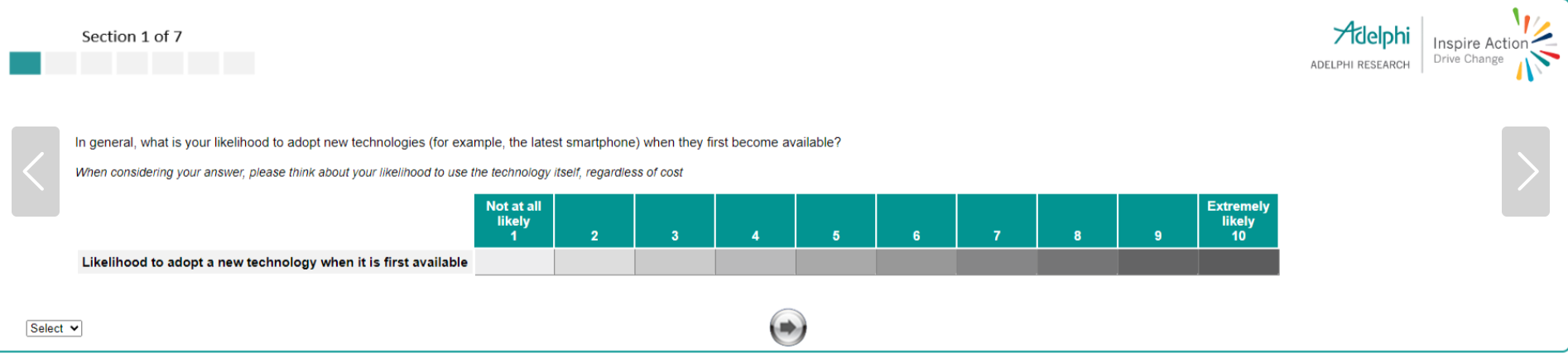


**S9. Attributes removed based on qualitative feedback from MS patients.**

- Injection site attribute was removed since a number of patients reported that this attribute was not important or would not impact or have an influence on device choice decision making.
- Skin reaction attribute was removed since, in addition to not being impactful or influential to some patients, further investigation indicated that skin reaction may not be significantly different between the types of devices and features and may be confounded with the very subjective nature of pain perception during or after an injection.
- Device type and duration A&Ls were retained since the preferences on the attribute levels are a key decision factor for device development. Additionally, participants reporting this attribute as not being important, impactful or influential in device choice may be related to the difficulty reported in the understanding of the attribute and associated levels. The latter will be addressed by providing further explanation in the educational material accompanying the DCE survey.
- All remaining A&Ls were retained for the DCE as each were considered by patients to be important and influential to device decision-making: handling steps, site of care and injection administration and injection frequency.

|  |  |  |  |  |  |  |  |  |  |  |  |  |  |  |  |  |  |  |  |  |  |  |  |  |  |  |  |  |
| --- | --- | --- | --- | --- | --- | --- | --- | --- | --- | --- | --- | --- | --- | --- | --- | --- | --- | --- | --- | --- | --- | --- | --- | --- | --- | --- | --- | --- |
